# Regional PM_2.5_ pollution forecasting using a hybrid model based on multi-scales feature fusion and deep learning algorithms

**DOI:** 10.1371/journal.pone.0333489

**Published:** 2025-10-09

**Authors:** Yong Zhang, Wenya Zhang, Bo Wu, Weichen Yi

**Affiliations:** 1 College of Mathematics and Statistics, Jishou University, Jishou, China; 2 School of Statistics and Mathematics, Zhongnan University of Economics and Law, Wuhan, China; 3 School of Rehabilitation Medicine and Health, Hunan University of Medicine, Huaihua, China; Universita degli Studi del Molise, ITALY

## Abstract

The issue of regional haze pollution has become increasingly prominent. However, early warning models for regional haze pollution are significantly lacking. To accurately predict regional PM_2.5_ pollution, hourly average concentration data of pollutants from January 1, 2021, to December 31, 2023 in the Chengdu-Chongqing urban agglomeration, along with concurrent surface meteorological data, are used and builds multi-scales feature fusion regional pollution prediction network (MSFRPM) based on a multi-input-multi-output deep learning framework. This model can simultaneously forecast PM_2.5_ concentrations for all cities in the region. The results show that the annual and seasonal prediction evaluation metrics of the MSFRPM model are significantly better than those of the baseline models. This can be attributed to the ability of the MSFRPM model to effectively capture the temporal dependency of historical PM_2.5_, the complex nonlinear relationships between other pollutants and meteorological factors within cities, and the multi-scales spatiotemporal dependencies of PM_2.5_ transport between cities in the urban agglomeration. In 2023, the Chengdu-Chongqing urban agglomeration experienced 15 days of mild regional pollution, 21 days of moderate pollution, and 2 days of severe pollution, with moderate pollution being the dominant type of PM_2.5_ pollution. Seasonally, regional PM_2.5_ pollution in the Chengdu-Chongqing urban agglomeration is mainly concentrated in the winter. The MSFRPM model assesses that the interannual and seasonal assessments of regional PM_2.5_ pollution in the Chengdu-Chongqing urban agglomeration in 2023 are generally consistent with actual observations. Accurate prediction of regional PM_2.5_ pollution is of great significance for the coordinated management and early warning of regional pollution.

## 1. Introduction

With the comprehensive implementation and promotion of the Airborne Pollution Action Plan, PM_2.5_ pollution in China has significantly improved [1–3]. PM_2.5_ concentrations in the Beijing-Tianjin-Hebei, Yangtze River Delta, Pearl River Delta, and Chengdu-Chongqing urban agglomerations have also simultaneously decreased [4]. However, looking only at changes in ρ(PM_2.5_) cannot fully reflect the trend of regional PM_2.5_ pollution. Haze pollution in urban agglomerations has shown significant regional characteristics. These regional PM_2.5_ pollution not only reduce atmospheric visibility but, more importantly, pose serious threats to the physical and mental health of urban residents [5]. The numerical prediction of the evolution of regional PM_2.5_ pollution is not only of great academic significance but also a pressing need for the current society, holding significant research value. It has already become a key focus in the field of atmospheric environment research.

Air pollution events refer to the phenomenon where pollutants emitted by human activities exceed concentration limits after undergoing a series of physical and chemical processes in the atmosphere [6–7]. Regional pollution is mainly primarily the result of local emissions and cross-regional transport interacting under unfavorable weather environments. When emissions from regional pollution sources are relatively stable, the concentration of pollutants mainly depends on the atmospheric diffusion capacity, closely related to surface weather patterns and meteorological factors [8]. Variations in regional meteorological conditions have a direct impact on the accumulation of pollutants, as well as the formation and dissipation of severe pollution. During periods of large-scale stable weather, pollutants released from human activities accumulate and transform rapidly, leading to a quick increase in local pollution concentrations, and through transport and mixing, form large-scale, high-concentration pollution air masses, which is a typical triggering mechanism for regional pollution events [9–11]. Currently, Various methods have been used to predict air pollution events, such as chemical transport models, statistical methods, and deep learning. Chemical transport models characterize the impact of emissions and meteorological conditions on air quality by adjusting emission inventories and meteorological settings, and designing multiple scenario analyses [12–13]. However, as a numerical simulation, chemical transport models have relatively high demands for computer resources. Additionally, the uncertainty in emission inventories and meteorological data somewhat diminishes the reliability of the analysis results. Traditional statistical methods are developed based on linear assumptions, assuming that the relationships between variables are linear. However, in real atmospheric systems, most time series of air pollution concentrations exhibit significant non-linear and non-stationary characteristics [14–16]. This makes traditional statistical methods perform poorly in predicting air quality time series. Deep learning is a machine learning method that employs multi-layer deep neural network structures, where neural networks consist of a large number of interconnected neurons, and information is transmitted from one layer of neurons to the next through activation functions [17,18]. Deep learning can efficiently massive and complex air quality data, leveraging stronger feature extraction and representation capabilities to capture the inherent patterns and complex relationships contained in air quality time series [19–27]. Current studies focusing on air quality prediction using deep learning have achieved substantial advancements but have mainly focused on predicting PM_2.5_ pollution in individual cities [28–36]. However, in recent years, PM_2.5_ pollution has expanded beyond single cities to exhibit regional characteristics [37]. Since the formation of regional pollution processes is closely related to weather patterns, differences in meteorological conditions and pollutant transport can lead to significant variations in the impact range and evolution of pollution events, which poses significant challenges for assessment and prediction at the regional scale. Therefore, traditional predictions of PM_2.5_ for individual cities can no longer address the current challenges of regional PM_2.5_ pollution forecasting. Constructing effective deep learning predictive models that incorporate regional pollution characteristics has become a primary task for current regional pollution forecasting.

The Chengdu-Chongqing urban agglomeration is one of the most urbanized regions in western China and is identified as a key area for air pollution control under the “Three Zones and Ten Regions” initiative [38]. The Sichuan Basin is encircled by mountains, and its deep basin topography makes it highly susceptible to prolonged stable weather conditions in winter, resulting in poor vertical and horizontal diffusion conditions. The prolonged accumulation of pollutants, along with the input from the southern Sichuan region, makes regional pollution events likely to occur. According to the 2023 Sichuan Province Environmental Air Quality Annual Report, 11 cities in the Sichuan Basin still had annual average PM_2.5_ concentrations exceeding the national secondary standard for ambient air quality (35 µg/m^3^), and PM_2.5_ pollution has not been fully controlled. Therefore, building a regional PM_2.5_ prediction model based on the Chengdu-Chongqing urban agglomeration has significant practical implications for preventing regional pollution in this area.

## 2. Data and methods

### 2.1. Data

The Chengdu-Chongqing urban cluster, with Chengdu and Chongqing at its core, constitutes a key economic and cultural center in western China, hosting a resident population of nearly 100 million. Geographically, the urban agglomeration spans approximately 101°57′-108°56′E and 27°40′-32°19′N, covering a total area of about 185,000 km^2^. The air pollutant monitoring data includes hourly average concentration data for 16 cities in the Chengdu-Chongqing urban agglomeration (Chengdu, Zigong, Luzhou, Deyang, Mianyang, Suining, Neijiang, Leshan, Nanchong, Meishan, Yibin, Guang’an, Dazhou, Ya’an, Ziyang, and Chongqing), with the study period from January 1, 2021, to December 31, 2023. The primary pollutants monitored are SO_2_, NO_2_, CO, PM_2.5_, PM_10,_ and O_3_. The air pollution data is sourced from the National Urban Air Quality Real-time Release Platform (https://www.aqistudy.cn/). Missing pollution data was filled using the average of adjacent values. In addition, meteorological data for the same period comes from the China Meteorological Data Service Center (http://data.cma.cn/), primarily including meteorological factors such as temperature, relative humidity, atmospheric pressure, rainfall, and wind speed. Missing values in the datasets for each indicator were supplemented using the cubic spline interpolation method.

### 2.2. Methods

#### 2.2.1. Data preprocessing.

To better capture the dependency features within different variables, this paper classifies the input data of the MSFRPM model into three categories: historical PM_2.5_ concentrations, exogenous variables (SO_2_, NO_2_, CO, PM_10_, and O_3_ and meteorological data) data, and PM_2.5_ data from various cities in the region. It is worth noting that the correlation relationships between the three categories of input data and future PM_2.5_ concentrations are not consistent. For example, historical PM_2.5_ concentrations data has a temporal dependency relationship with future PM_2.5_ concentrations, while exogenous variables show a nonlinear dependency relationship, and PM_2.5_ data from various cities in the region has a spatial dependency relationship with the future PM_2.5_ concentrations in the target city. However, different dependency features require different deep learning structures for processing and mapping. Accordingly, to more accurately capture the dependency relationships of the input variables, this paper needs to adjust the input data. The input of historical PM_2.5_ concentrations are feed to LSTM models and exogenous variables follow the conventional deep neural network (DNN). For the PM_2.5_ data from various cities in the region, we adopt the preprocessing method proposed by Xiao et al. [39], which transforms the sequence data into a graph structure, which is more conducive for the multi-scales pyramid network to capture the multi-scales spatial dependency characteristics of pollutant transport.

To mitigate the impact of different indicator scales and enhance model efficiency, all input dataset should have been processed by normalization as follow:


$$X^{\prime }=\frac{X-X_{\min}}{X_{\max}-X_{\min}}$$ (1)

where, $X^{\prime }$ is the normalized data for each indicator, with a range of 0 to 1. X refers to the original data values for each indicator. $X_{\min}$ indicates the minimum value. $X_{\max}$ represents the maximum value.

#### 2.2.2. Definition of regional PM_2.5_ pollution.

This study refers to the “Technical Guide for the Evaluation of Ambient Air Quality Forecasting Performance” regarding the definition of regional pollution levels, using five adjacent prefecture-level or higher cities as the spatial scope for determining regional pollution [40]. This definition is consistent with the HJ 633 − 2012 “Ambient Air Quality Index (AQI) Technical Regulation”, using natural days as the time unit for determining regional pollution, a regional PM_2.5_ pollution definition and classification system for the Yangtze River Delta region was developed [41]. Mild regional pollution: In one natural day, more than five contiguous cities within the region experience PM_2.5_ pollution, with no more than four cities classified as moderately polluted, and the rest experiencing mild pollution. Moderate regional pollution: In one natural day, more than five contiguous cities in the region experience PM_2.5_ pollution, with no more than four cities classified as heavily polluted or worse; if no cities experience heavy pollution or worse, at least five cities must be moderately polluted. Severe regional pollution: Within a single natural day, more than five contiguous cities in the region exhibit PM_2.5_ pollution, with at least five cities reaching severe or higher pollution levels.

#### 2.2.3. Multi-scales feature fusion regional pollution prediction network.

**Fig 1:**
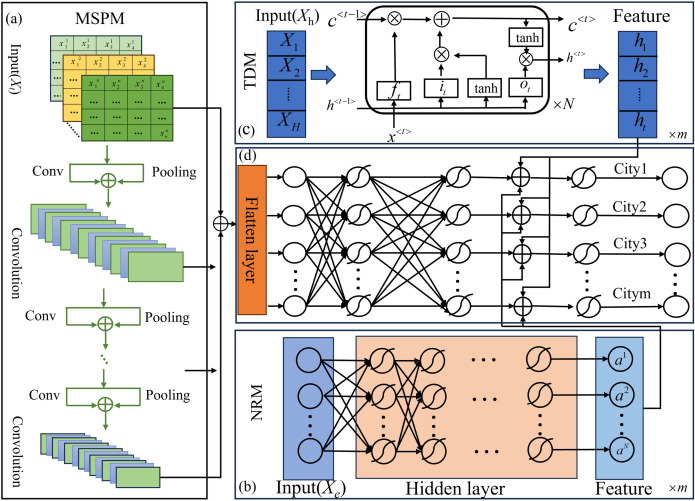
The overall framework of MSFRPM.


In the atmospheric system, the evolution of PM_2.5_ concentrations in a city is influenced not only by its historical PM_2.5_ values, exogenous variables, but also by regional transport [42]. However, the patterns of influence on PM_2.5_ may vary significantly across different factors. The historical values of PM_2.5_ primarily have a temporal dependency relationship with future PM_2.5_, while exogenous variables mainly present nonlinear dependencies. Regional transport of pollutants plays a crucial role in the formation and maintenance of air pollution events [11,43]. For emission source areas, it is generally believed that stagnant meteorological conditions such as weak surface winds, strong temperature inversions, subsidence, and low mixing layer heights are conducive to the accumulation of air pollutants, leading to heavy air pollution [44,45]. Under unique non-stagnant meteorological conditions of strong near-surface winds, no temperature inversion, and additional instability in the atmospheric boundary layer, regional transport of PM_2.5_ exacerbates regional pollution [46,47]. Governed by strong winds in the lower troposphere and vertical diffusion, air pollutants can be transported over long distances from the pollution source to downwind receptor areas, significantly expanding the affected region [48,49]. Compared to source-area pollutants, the impact of pollutant transport is also not negligible. It is worth noting that the impact of regional transport on PM_2.5_ evolution of a city is primarily spatially dependent. Due to the varying distances between different transport sources and the target city, the timing of transport shows significant differences. This results in significant differences in the spatial dependency of regional transport at different time scales. If only traditional methods are used, assuming consistent dependency relationships among variables influencing PM_2.5_ evolution, significant errors may occur in PM_2.5_ concentrations predictions. To address this, this paper uses a locally connected approach to design a multi-scales deep learning module for the spatial dependencies at different time scales, ultimately outputting accurate predictions through a fully connected layer. The detailed process is as follows:

Step 1: For PM_2.5_ historical values ($X_{h}$), the relationship between this data and PM_2.5_ concentrations is primarily a temporal dependency relationship. In deep learning, LSTM effectively learns long-term dependencies in time series using a unique feedback structure and gating mechanism [50]. Therefore, a temporal dependency module (TDM) is built using the LSTM model to capture the temporal dependencies between $X_{h}$ and future PM_2.5_ values, as illustrated in Fig 1c. The specific process is as follows:

$$f_{t}=\sigma (W_{f}X_{h}+U_{f}h_{t-1}+b_{f})$$ (2)

$$i_{t}=\sigma (W_{i}X_{h}+U_{i}h_{t-1}+b_{i})$$ (3)

$$o_{t}=\sigma (W_{o}X_{h}+U_{o}h_{t-1}+b_{o})$$ (4)

$${\tilde{c}}_{t}=\sigma (W_{c}X_{h}+U_{c}h_{t-1}+b_{c})$$ (5)

$$c_{t}=f_{t}\circ c_{t-1}+i_{t}\circ {\tilde{c}}_{t}$$ (6)

$$h_{t}=o_{t}\circ \tanh(c_{t})$$ (7)

where, $X_{h}\in R^{D}$ represents the historical value of PM_2.5_ concentrations. $f_{t}\in R^{D}$ indicates the forget gate. $o_{t}\in R^{D}$ is the output gate. $i_{t}\in R^{D}$ indicates the input gate. $h_{t}\in R^{D}$ refers to the feature vector of temporal dependencies. ${\tilde{c}}_{t}\in R^{D}$ indicates a neuron. $c_{t}\in R^{d}$ represents a memory neuron. $\sigma_{g}$ is the sigmoid activation function. $W(\cdot )\in R^{D\times d}$, $U(\cdot )\in R^{D\times d}$ and $b(\cdot )\in R^{D\times d}$ represent the weight and bias matrices. D represents the spatial dimension. ◦ represents the Hadamard product.

Step 2: For the complex nonlinear relationship between exogenous variables ($X_{e}$) and PM_2.5_ concentrations, a deep neural network (DNN) framework is used to capture the complex, nonlinear correlations between variables. The DNN possesses powerful feature learning and nonlinear processing capabilities. In the DNN, $X_{e}$ serves as the input data, fed into the DNN, undergoing layer-by-layer computations in the hidden layers and nonlinear transformations through the activation function, enabling the DNN to effectively extract complex nonlinear relationship features between other pollutants, meteorological factors, and PM_2.5_ concentrations. Accordingly, a nonlinear relationship module (NRM) is further constructed based on the DNN structure, with its structure shown in Fig 1b. The calculation formula for the NRM module is as follows:


$$a_{j}^{\text{1}}=\sigma \left(\sum_{k=1}^{n_{l}-1}w_{j,k}^{\text{1}}X_{e}+b_{j}^{\text{1}}\right)j=1,2,\cdots ,m_{l}$$ (8)

$$a_{j}^{l}=\sigma \left(\sum_{k=1}^{n_{l}-1}w_{j,k}^{l}a_{k}^{l-1}+b_{j}^{l}\right),l=2,3,\cdots ,N-1,j=1,2,\cdots ,m_{l}$$ (9)

$$a^{l}=\sigma \left(W^{l}a^{l-1}+B^{l}\right),l=2,3,\cdots ,N-1$$ (10)

where, $X_{e}$ represents the exogenous variables within the city, such as pollutants like SO_2_, NO_2_, CO, PM_10_, and O_3_, as well as meteorological factors. l=1,2,3,⋯,N, where l=1 and l=N represent the input layer and output layer, respectively. l=2,3,⋯,N−1 represents the hidden layers. $a_{j}^{l}$ represents the nonlinear feature of the j -th neuron in the l -th layer after the activation function σ. $W^{l}$ and $B^{l}$ denote the weight and bias vectors. $a^{l}$ represents the feature vector of the nonlinear dependency of the exogenous variables.

Step 3: To effectively extract the influence of the spatial dependencies of regional transport on PM_2.5_ concentrations at different time scales, a module for extracting multi-scales spatial features (MSPM) has been designed based on a multi-scales pyramid network, illustrated in Fig 1a. According to the pyramid network structure, multiple convolutional layers are utilized to convert the original time series into feature representations hierarchically, from smaller scales to larger scales. This multi-scales structure provides us with the opportunity to observe the original time series at different time scales. Specifically, feature representations at smaller scales can retain spatial features of transport over shorter distances, while feature representations at larger scales can capture spatial features of transport over longer distances. The computation process of MSPM is as follows:


$$X^{\text{1}}=ReLU(W^{\text{1}}⊗X_{t}+b^{\text{1}})$$ (11)

$$X^{k}=ReLU(W^{k}⊗X^{k-1}+b^{k})$$ (12)

$$X_{norm}^{k}=Pooling(ReLU(W_{norm}^{k}⊗X^{k-1}+b_{norm}^{k}))$$ (13)

$$MF^{k}=X^{k}+X_{norm}^{k}$$ (14)

where, ⊗ represents the convolution operator. $X_{t}$ denotes the PM_2.5_ concentrations values of various cities within the region. $W^{\text{1}}$ and $b^{\text{1}}$ denote the weights and biases for the first convolution operation. $W^{k}$ and $b^{k}$ represent the convolution kernel and bias vector of the *k*-th layer, respectively. ReLU denotes the ReLU activation function. Pooling is the pooling operation. $MF^{k}(k=1,2,\cdots ,N)$ represents the multi-scales spatial features.

Step 4: Finally, to achieve regional pollution prediction, a deep learning multi-output structure is employed to simultaneously output PM_2.5_ pollution for all cities in the urban agglomeration, enabling regional pollution assessment. The multi-output structure mainly consists of two layers: one is the feature fusion layer, and the other is the multi-output layer, as shown in Fig 1d. The structural formulas are as follows:


$$F=W^{\text{1}}h_{t}+W^{\text{2}}a^{l}+W^{\text{3}}MF^{k}+b$$ (15)

$$\left[y_{t+w}^{\text{1}},y_{t+w}^{\text{2}},\cdots ,y_{t+w}^{m}]=\sigma (F_{1:t}\right)$$ (16)

where, $W^{\text{1}}$, $W^{\text{2}}$, $W^{\text{3}}$ and b denote the weights and biases for the historical dependency features of PM_2.5_, the nonlinear features of exogenous variables, and the multi-scales spatial features of pollution transport, respectively. σ() represents the sigmoid activation function. $y_{t+w}^{m}$ represents the predicted PM_2.5_ value for the m -th city in the urban cluster. m represents the number of cities within the urban agglomeration.

#### 2.2.4. Loss function.

As MSFRPM is a multi-output deep learning model, conventional loss functions are inadequate for optimizing the MSFRPM model. To enable gradient descent-based optimization algorithms to be applicable to the MSFRPM model, a multi-output loss function (MOLF) is proposed. The specific formula for MOLF is as follows:


$$MOLF=\frac{\text{1}}{NM}\sum_{i=1}^{M}\sum_{j=1}^{N}{\left(y_{ij}-{\hat{y}}_{ij}\right)}^{\text{2}}$$

where M denotes the number of cities in city clusters. N denotes the length of the PM_2.5_ series. $y_{ij}$ represents the observed value of i city at timej. ${\hat{y}}_{ij}$ represents the predicted value of i city at timej.

#### 2.2.5. Experimental setup.

In the experiment, we split the preprocessed data into training, validation, and test sets in a ratio of 7:1:2. The first 70% of the data was used as the training set, the next 10% as the validation set, and the remaining 20% as the test set. We employed the MSFRPM prediction model, with an input window length of 168 (one week) and an output prediction length of 1. The number of training batches is set to 30, with the learning rate set at 0.0001. The maximum number of training epochs is 100. The optimizer used for the MSFRPM is Adam, and the loss function is MOLF loss function.

#### 2.2.6. Evaluation metrics.

To assess performance of the proposed multi-scales feature fusion regional pollution prediction model, the ten-fold cross-validation algorithm are employed in this paper, which can effectively prevent model overfitting [51]. The steps of the ten-fold cross-validation algorithm are as follows: First, the input data set of the model is randomly divided into 10 subsets of equal length. Then, the model is trained 10 times, with each training using one of the subsets (without repetition) as the test set, while the remaining 9 subsets serve as the training set. Finally, the model performance is evaluated. In evaluating the model, R^2^, Mean Absolute Error (MAE), and Root Mean Square Error (RMSE) are selected as the three metrics to assess the performance of model. The R^2^ value ranges from [0, 1]; the closer the value is to 1, the better the model performance, and the closer it is to 0, the worse the performance. MAE and RMSE mainly measure the error between the predicted and actual values, and the closer their values are to 0, the better the model performance. The formulas for the evaluation metrics are as follows:


$$R^{\text{2}}=1-\frac{\sum_{i=1}^{n}{\left(y_{i}-{\hat{y}}_{i}\right)}^{\text{2}}}{\sum_{i=1}^{n}{\left(y_{i}-{\overset{―}{y}}_{i}\right)}^{\text{2}}}$$ (17)

$$MAE=\frac{\text{1}}{N}\sum_{i=1}^{n}\left|y_{i}-{\hat{y}}_{i}\right|$$ (18)

$$RMSE=\sqrt{\frac{\text{1}}{N}\sum_{i=1}^{n}{\left(y_{i}-{\hat{y}}_{i}\right)}^{\text{2}}}$$ (19)

where, N denotes the length of the pollutant series. $y_{i}$ refers to the PM_2.5_ concentrations value at time i. ${\hat{y}}_{i}$ represents the predicted PM_2.5_ concentrations at time i. ${\overset{―}{y}}_{i}$ are the average values of PM_2.5_ concentrations.

## 3. Results and discussion

### 3.1. Statistical analysis

Table 1 shows the basic statistics of PM_2.5_ pollution concentrations data in the Chengdu-Chongqing urban agglomeration. As shown in the Table 1, the maximum values of PM_2.5_ concentrations in each city of the Chengdu-Chongqing urban agglomeration in 2023 was 201.000 µg/m^3^, 204.000 µg/m^3^, 215.000 µg/m^3^, 226.000 µg/m^3^, 207.000 µg/m^3^, 214.000 µg/m^3^, 226.000 µg/m^3^, 218.000 µg/m^3^, 205.000 µg/m^3^, 234.000 µg/m^3^, 195.000 µg/m^3^, 240.000 µg/m^3^, 209.000 µg/m^3^, 209.000 µg/m^3^, 316.000 µg/m^3^, and 169.000 µg/m^3^, respectively. The maximum values for all cities are far higher than the National Ambient Air Quality Standard (GB 3095−2012), which stipulates a PM_2.5_ annual concentration limit of 35 µg/m^3^ for the secondary standard. The average values of PM_2.5_ concentrations in the cities of the Chengdu-Chongqing urban agglomeration in 2023 were 39.297 µg/m^3^, 41.619 µg/m^3^, 36.981 µg/m^3^, 38.318 µg/m^3^, 35.005 µg/m^3^, 30.069 µg/m^3^, 40.673 µg/m^3^, 32.012 µg/m^3^, 43.057 µg/m^3^, 43.853 µg/m^3^, 40.092 µg/m^3^, 36.572 µg/m^3^, 44.235 µg/m^3^, 31.397 µg/m^3^, 40.369 µg/m^3^, and 36.585 µg/m^3^, respectively. The average values in most cities exceeded the national primary PM_2.5_ standard limit of 35 µg/m^3^, indicating that haze pollution in the Chengdu-Chongqing urban agglomeration is still very serious, and most cities simultaneously exceeded the national primary PM_2.5_ standard limit of 35 µg/m^3^. This suggests that PM_2.5_ pollution in the Chengdu-Chongqing urban agglomeration has clearly developed into a regional pollution issue. The standard deviation of each city was significantly greater than 0, indicating that the PM_2.5_ concentrations fluctuations in each city of the Chengdu-Chongqing urban agglomeration are quite severe. Moreover, based on skewness and kurtosis, the PM_2.5_ series deviates from a normal distribution. The skewness values are notably greater than 0, suggesting that most PM_2.5_ concentrations are above the mean. Further applying the Lilliefors test to the PM_2.5_ series showed that the Lilliefors statistics were markedly higher than the critical value (0.013). This indicates that the null hypothesis is rejected in the Lilliefors test, showing that the PM_2.5_ concentrations sequences in each city of the Chengdu-Chongqing urban agglomeration in 2023 do not follow a normal distribution and exhibit significant nonlinear characteristics. This also suggests that the evolution of regional PM_2.5_ pollution in the Chengdu-Chongqing urban agglomeration may be influenced by nonlinear dynamic mechanisms, and that traditional linear statistical prediction methods may not meet the requirements for regional pollution assessment.

**Table 1:** Basic statistical metrics of PM_2.5_ pollution concentration.

Cities	Max (μg/m^3^)	Min (μg/m^3^)	Mean (μg/m^3^)	Std (μg/m^3^)	Skewness (μg/m^3^)	Kurtosis (μg/m^3^)	Lilliefors (P-values)
Chengdu	201.00	1.00	39.30	30.85	1.74	3.66	0.00
Deyang	204.00	2.00	41.61	32.54	1.73	3.44	0.00
Mianyang	215.00	2.00	36.98	27.89	1.82	4.45	0.00
Meishan	226.00	1.00	38.31	34.51	1.72	3.46	0.00
Ziyang	207.00	1.00	35.00	30.52	1.72	3.18	0.00
Suining	214.00	2.00	30.06	26.83	1.85	4.3	0.00
Leshan	226.00	1.00	40.67	31.37	1.69	3.32	0.00
Ya’an	218.00	1.00	32.01	27.29	2.10	6.75	0.00
Zigong	205.00	2.00	43.05	34.29	1.45	1.94	0.00
Luzhou	234.00	1.00	43.85	37.32	1.65	3.04	0.00
Neijiang	195.00	2.00	40.09	31.82	1.61	2.61	0.00
Nanchong	240.00	1.00	36.57	28.12	1.83	4.83	0.00
Yibin	209.00	1.00	44.23	33.57	1.46	2.24	0.00
Dazhou	209.00	1.00	31.39	32.53	2.19	5.79	0.00
Guang’an	316.00	2.00	40.36	35.36	2.21	6.89	0.00
Chongqing	169.00	1.00	36.58	26.61	1.78	3.58	0.00

Furthermore, based on the definitions of regional pollution from the “Technical Guidelines for Evaluating the Effectiveness of Ambient Air Quality Forecasts” and the “Technical Specifications for Ambient Air Quality Index (AQI)” the regional PM_2.5_ pollution in the Chengdu-Chongqing urban agglomeration in 2023 was statistically analyzed. Annually, in 2023, the Chengdu-Chongqing urban agglomeration experienced 15 occurrences of mild regional pollution, 21 occurrences of moderate regional pollution, and 2 occurrences of severe regional pollution. Annually, in 2023, the Chengdu-Chongqing urban agglomeration experienced 15 occurrences of mild regional pollution, 21 occurrences of moderate regional pollution, and 2 occurrences of severe regional pollution events. This result indicates that moderate regional pollution is predominant in PM_2.5_ pollution within the Chengdu-Chongqing urban agglomeration. In terms of seasons, during spring 2023, the Chengdu-Chongqing urban agglomeration experienced 1 occurrence of mild regional pollution, 0 occurrences of moderate regional pollution, and 0 occurrences of severe regional pollution events. In summer, the Chengdu-Chongqing urban agglomeration experienced 0 occurrences of mild regional pollution, 0 occurrences of moderate regional pollution, and 0 occurrences of severe regional pollution events. In autumn, the Chengdu-Chongqing urban agglomeration experienced 1 occurrence of mild regional pollution, 0 occurrences of moderate pollution, and 0 occurrences of severe pollution events. In winter, the Chengdu-Chongqing urban agglomeration experienced 14 occurrences of mild regional pollution, 21 occurrences of moderate regional pollution, and 2 occurrences of severe regional pollution events. These results indicate that mild, moderate, and severe regional pollution mainly occurred in winter.

### 3.2. Model validation

#### 3.2.1. Model training and testing.

To achieve optimal performance of the MSFRPM model, this study primarily adjusts two important hyperparameters: the size of the convolutional kernel and the length of the input data. In the tuning process, the convolutional kernel sizes were set to 2, 4, 6, 8, and 10, and the input data lengths were set to 4, 12, 24, 120, and 168. It was found through experiments that the model achieved optimal performance with a convolutional kernel size of 4 and an input length of 24. The results are presented in Table 2, showing an average MAE of 4.04 µg/m^3^, an average R^2^ of 0.97, and an average RMSE of 6.13 µg/m^3^ for all cities.

**Table 2:** Comparison results of predictions between MSFRPM model and benchmark models.

Models	MAE(μg/m^3^)	R^2^	RMSE(μg/m^3^)
XGBoost	8.49	0.89	12.10
SVM	9.26	0.91	10.94
RF	4.35	0.97	6.28
BPNN	5.06	0.97	6.27
LSTM	6.53	0.93	9.96
ConvLSTM	4.43	0.97	5.77
MSFRPM(Chengdu)	3.70	0.98	5.09
MSFRPM(Regional)	4.04	0.97	6.13

#### 3.3.2. Annual model performance.

**Fig 2:**
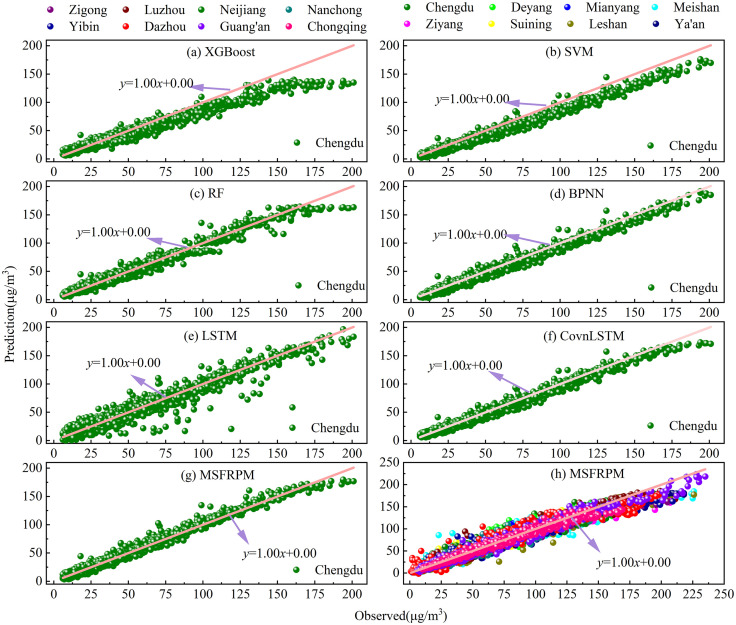
Prediction results of PM_2.5_ concentrations by XGBoost, SVM, RF, BPNN, LSTM, ConvLSTM, and MSFRPM Models.

To assess the validity of the MSFRPM model, this section conducts a comparative analysis using benchmark models including XGBoost, SVM, RF, BPNN, LSTM, and ConvLSTM. Fig 2 illustrates the prediction results of PM_2.5_ concentrations predictions for the XGBoost, SVM, RF, BPNN, LSTM, ConvLSTM, and MSFRPM models in 2023. The R^2^ values for XGBoost, SVM, and LSTM were relatively low, whereas RF, BPNN, ConvLSTM, and MSFRPM models all surpassed an R^2^ of 0.97. The three metrics for deep learning models (excluding LSTM) showed significant improvements over traditional machine learning models like XGBoost, SVM, and RF. This improvement may primarily be due to the ability of deep learning models to better capture the nonlinear relationships between PM_2.5_ concentrations and other factors. The poor performance of LSTM may be related to its structure, as LSTM primarily explores the inherent temporal dependencies in time series, while the fluctuations in PM_2.5_ concentration in the Chengdu-Chongqing urban agglomeration are less influenced by residual particles in the atmospheric system, leading to poorer performance. Compared to deep models, MSFRPM takes into account the complex nonlinear relationships among various precursors and their meteorological factors within cities, as well as the spatial dependency relationships of PM_2.5_ concentrations across different scales among cities in the Chengdu-Chongqing region, yielding an MAE of 3.70 µg/m^3^, an R^2^ of 0.98, and an RMSE of 5.09 µg/m^3^.

Furthermore, based on the fitting graph, it can be observed that when PM_2.5_ concentrations is low, the fitting line of predicted values and monitored values is very close to the theoretical fitting line (y = x), indicating that all prediction models perform well when PM_2.5_ concentrations are low. When PM_2.5_ concentrations are high, the fitting line of predicted and monitored values increasingly deviates from the theoretical fitting line as the concentration rises. This indicates that the performance of each prediction model declines as PM_2.5_ concentrations rise. Notably, regardless of how PM_2.5_ concentrations change, the scatter points for predicted and monitored values from the MSFRPM model are evenly distributed around the theoretical fitting line. These results indicate that the MSFRPM model consistently outperforms XGBoost, SVM, RF, BPNN, LSTM, and ConvLSTM models, whether pollution levels are low or high. This is primarily because the MSFRPM model takes into account not only the temporal dependencies of historical PM_2.5_ values but also the complex nonlinear relationships between other pollutants and meteorological factors within cities, as well as the spatial dependencies of PM_2.5_ concentrations at different scales among cities in the city cluster. However, when concentrations exceed 190 µg/m³, the MSFRPM model tends to underestimate. This phenomenon may primarily result from unmeasurable anthropogenic emission factors.

#### 3.2.3. Seasonal performance of MSFRPM model.

**Fig 3:**
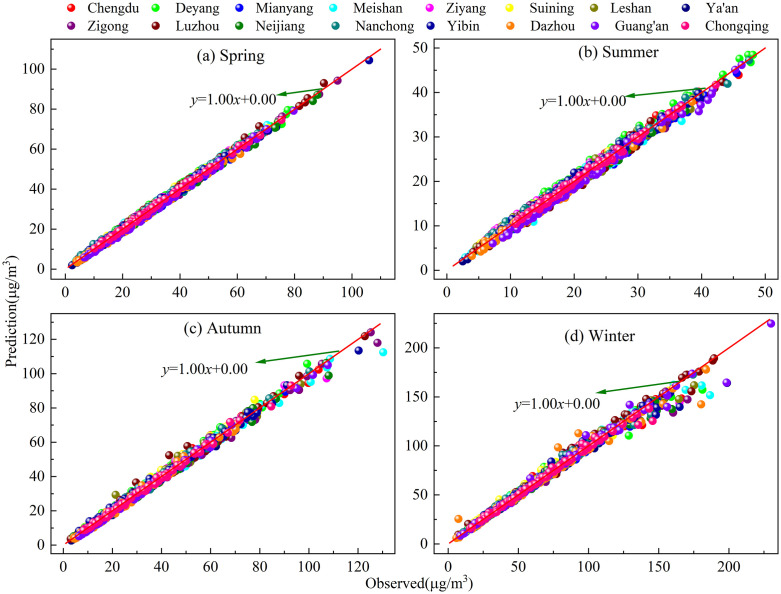
Seasonal predictions result of PM_2.5_ concentrations using the MSFRPM model.

Fig 3 shows the prediction results of seasonal PM_2.5_ concentrations predictions by the MSFRPM model in the Chengdu-Chongqing urban agglomeration. Due to the differences in the evolution patterns of PM_2.5_ concentrations across seasons, the predictive performance of the MSFRPM model also varies. The MAE for the MSFRPM model in spring, summer, autumn, and winter are 2.63 µg/m^3^, 2.10 µg/m^3^, 2.82 µg/m^3^, and 4.28 µg/m^3^, respectively; R^2^ values are 0.96, 0.91, 0.97, and 0.97; and RMSE values are 3.67 µg/m^3^, 2.94 µg/m^3^, 4.06 µg/m^3^, and 6.77 µg/m^3^, respectively. Although the MAE and RMSE are lower in summer, the R^2^ is also the lowest. The lower MAE and RMSE may be attributed to favorable atmospheric transport conditions and the cleansing effect of rainfall in summer. The lower R^2^ suggests that the predictive performance of the MSFRPM model is weakest in summer. Despite higher MAE and RMSE values in spring, autumn, and winter, the R^2^ values exceed 0.90 in all cases. The higher R^2^ indicates that the MSFRPM model can effectively capture the evolution patterns of PM_2.5_ concentrations across cities in the Chengdu-Chongqing urban agglomeration. This is mainly because the Chengdu-Chongqing urban agglomeration is located in the Sichuan Basin, where winter is characterized by calm winds, weak atmospheric transport capacity, and strong stable stratification, making it easier for the MSFRPM model to capture the evolution patterns of PM_2.5_ concentrations. The higher errors may be attributed to anthropogenic emissions, such as increased population mobility during winter holidays and higher emissions from firecrackers.

### 3.3. Comparison results of different machine learning algorithms

To evaluate the predictive performance of the MSFRPM model, this section further compares its PM_2.5_ predictions for Chengdu with those of XGBoost, SVM, RF, BPNN, LSTM, and ConvLSTM, as shown in Fig 2 and Table 2. According to the Table 2, the MSFRPM model exhibits the best predictive performance, followed by ConvLSTM, RF, BPNN, LSTM, XGBoost, and SVM. Compared to traditional machine learning methods (XGBoost, SVM, and RF), deep learning (excluding LSTM) shows better predictive performance for PM_2.5_. This is because deep learning models can better uncover the nonlinear relationships between PM_2.5_ concentrations and other factors. Compared to BPNN and LSTM, the ConvLSTM model exhibits improved predictive performance. This is due to the advantages of combining CNN and LSTM in the ConvLSTM model, which not only considers the temporal dependencies of PM_2.5_ concentrations but also incorporates the spatial dependencies among cities through the CNN structure. Compared to ConvLSTM, the MSFRPM model integrates the advantages of BPNN, CNN, and LSTM, taking into account the temporal dependencies of PM_2.5_ historical values, the complex nonlinear relationships between other pollutants and meteorological factors within cities, and the multi-scales spatiotemporal dependencies of PM_2.5_ concentrations across different cities in the urban agglomeration. Therefore, the MSFRPM model achieves optimal prediction results for PM_2.5_ in the Chengdu-Chongqing urban agglomeration.

### 3.4. Assessment of regional PM_2.5_ pollution concentration

#### 3.4.1. Annual variation of regional PM_2.5_ pollution concentration.

**Fig 4:**
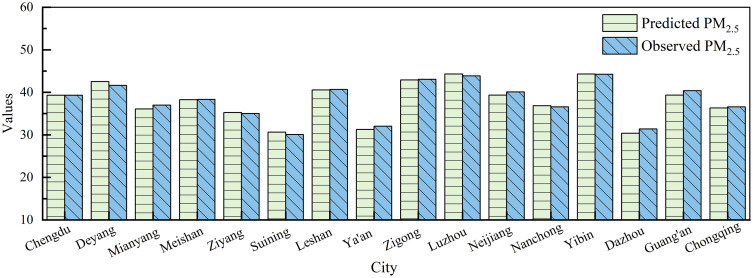
PM_2.5_ Concentrations distribution in the Chengdu-Chongqing urban agglomeration in 2023.

The spatiotemporal evaluation of PM_2.5_ for the Chengdu-Chongqing Urban Agglomeration in 2023 using the MSFRPM model is presented in Fig 4. Fig 4 indicates that the annual average PM_2.5_ values among cities in the Chengdu-Chongqing urban agglomeration exhibit notable differences, ranging from 30.385 µg/m^3^ to 44.306 µg/m^3^, with an average of 37.980 µg/m^3^. The actual monitored average ranged from 30.070 µg/m^3^ to 44.236 µg/m^3^, with a mean of 38.133 µg/m^3^. This result indicates a significant consistency between the MSFRPM assessment results and the fluctuations in the actual monitored concentration values.

According to Fig 4, the annual average PM_2.5_ pollution values in the Chengdu-Chongqing urban agglomeration exhibit significant spatial heterogeneity. The interannual PM_2.5_ pollution concentrations in the Chengdu-Chongqing urban agglomeration in 2023 can be roughly classified into four categories. The first category includes Yibin and Luzhou, with a concentration range of 43.4 to 48.0 μg/m^3^. The second category comprises Chengdu, Deyang, Leshan, Zigong, Neijiang, and Guang’an, with concentrations ranging from 38.80 to 43.40 μg/m^3^. The third category consists of Chongqing, Nanchong, Mianyang, Meishan, and Ziyang, with a range of 34.20 to 38.80 μg/m^3^. The fourth category includes Dazhou, Suining, and Ya’an, with a range of 29.60 to 34.2 μg/m^3^. The cities in the first category have higher annual average pollution concentrations, primarily because Yibin and Luzhou are major heavy industrial bases in the Chengdu-Chongqing urban agglomeration, where high-pollution industries such as petrochemicals, metal smelting, and cement manufacturing lead to severe pollution events. Within the second category, Zigong and Neijiang are significant industrial bases, and the pollution levels in Leshan are largely affected by PM_2.5_ transport from the northwest of Yibin and the south of Zigong [52,53]. The pollution in Chengdu and Deyang is mainly attributed to a combination of vehicle emissions in urban areas, high industrial emissions, pollutant transport from southern Sichuan, and locally unfavorable meteorological conditions.

#### 3.4.2. Seasonal variation of regional PM_2.5_ pollution concentration.

**Fig 5:**
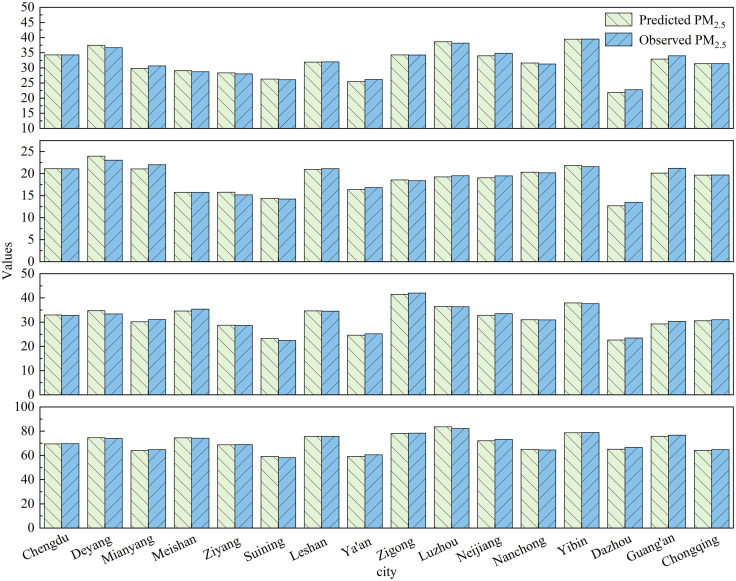
Distribution of PM_2.5_ concentrations in the Chengdu-Chongqing urban agglomeration during spring, summer, autumn, and winter.

Fig 5 illustrates the spatial distribution of PM_2.5_ concentrations in the Chengdu-Chongqing urban agglomeration across different seasons in 2023. As shown in Fig 5, PM_2.5_ concentrations exhibit a significant seasonal effect, characterized by lower levels in summer and higher levels in winter, with transitional patterns in spring and autumn. The average PM_2.5_ concentrations range for the cities in the Chengdu-Chongqing urban agglomeration during winter is 59.16 to 83.61 μg/m^3^, with significant pollution focused in southern Sichuan, mainly in Luzhou, Yibin, Zigong, and Neijiang. Southern Sichuan serves as a concentrated area for industrial bases within the Chengdu-Chongqing urban agglomeration, where high-pollution industries like petrochemicals, metal smelting, and cement manufacturing intensify pollution, affecting PM_2.5_ fluctuations in other cities through pollutant transport. The average PM_2.5_ concentrations range during summer is 12.70 to 23.95 μg/m^3^, which is significantly lower than the annual average for the urban cluster, primarily due to Chengdu’s humid and rainy summer conditions that facilitate wet deposition and diffusion of PM_2.5_. The average concentrations for spring and autumn are 31.67 μg/m^3^ and 31.61 μg/m^3^, respectively, with higher pollution levels concentrated mainly in southern Sichuan (Luzhou, Yibin, Zigong, and Neijiang) as well as in Chengdu and Deyang. The main reason is that southern Sichuan is a concentrated area for industrial bases in the Chengdu-Chongqing urban agglomeration, which exacerbates air pollution in the region.

#### 3.4.3. Assessment of regional PM_2.5_ pollution.

Furthermore, the evaluation of the frequency of regional PM_2.5_ pollution events in the Chengdu-Chongqing urban agglomeration using the MSFRPM model is generally consistent with the actual results, underestimating only one instance of mild regional pollution event and one instance of severe regional pollution event, as shown in Table 3. The MSFRPM model estimates that there was a total of 36 regional pollution events in the Chengdu-Chongqing urban agglomeration in 2023, including 14 instances of mild regional pollution events, 21 instances of moderate regional pollution events, and 1 instance of severe regional pollution event. In 2023, PM_2.5_ was primarily characterized by moderate regional pollution events, followed by mild regional pollution events. Regional pollution events exhibit notable differences among the seasons. During spring and autumn, there was only one instance of mild regional pollution event, while no regional pollution events occurred in summer. During winter, the Chengdu-Chongqing Urban Agglomeration recorded 12 instances of mild regional pollution events, 21 instances of moderate regional pollution events, and 1 instance of severe regional pollution event, with a higher occurrence of moderate regional pollution outbreaks. The primary reason for this is the combined effect of the geographical location of the Chengdu-Chongqing urban agglomeration and the winter climate. The Chengdu-Chongqing urban agglomeration is mainly located in the Sichuan Basin, where the near-surface atmospheric transport conditions are weak, making it difficult for air pollutants to disperse. These external conditions provide favorable conditions for the accumulation of PM_2.5_ pollution. During winter, the cities in the Chengdu-Chongqing Urban Agglomeration have high calm wind frequencies and low rainfall, which further diminish the ability to transport pollutants and enhance wet deposition, ultimately intensifying regional pollution.

**Table 3:** Regional PM_2.5_ pollution assessment results.

Classes	Year	Spring	Summer	Autumn	Winter
Mild regional pollution	14	1	0	1	12
Moderate regional pollution	21	0	0	0	21
Severe regional pollution	1	0	0	0	1

## 4. Conclusion

This study proposes multi-scales feature fusion regional pollution prediction network (MSFRPM) for accurate prediction of regional PM_2.5_ pollution, based on data from 16 cities in the Chengdu-Chongqing Urban Agglomeration from January 1, 2021, to December 31, 2023, including SO_2_, NO_2_, PM_10_, CO, O_3_, temperature, rainfall, pressure, relative humidity, and wind speed.

(1) The Chengdu-Chongqing urban agglomeration experienced mild regional pollution events for 15 days, moderate regional pollution events for 21 days, and severe regional pollution events for 2 days in 2023, indicating that PM_2.5_ pollution in the Chengdu-Chongqing urban agglomeration is primarily characterized by moderate regional pollution. From a seasonal perspective, PM_2.5_ in the Chengdu-Chongqing urban agglomeration is mainly concentrated in winter, while regional PM_2.5_ pollution is rarely observed in spring, summer, and autumn.
(2) The interannual prediction metrics of the MSFRPM model for 2023 are 3.70 μg/m^3^, with an R^2^ of 0.98 and an RMSE of 5.09 μg/m^3^. The MAE for each season is 2.63 μg/m^3^, 2.10 μg/m^3^, 2.82 μg/m^3^, and 4.28 μg/m^3^; the R^2^ values are 0.96, 0.91, 0.97, and 0.97; and the RMSE values are 3.67 μg/m^3^, 2.94 μg/m^3^, 4.06 μg/m^3^, and 6.77 μg/m^3^. This indicates that its predictive performance is significantly superior to that of models such as XGBoost, SVM, RF, BPNN, LSTM, and ConvLSTM.
(3) The interannual PM_2.5_ pollution concentrations in the Chengdu-Chongqing urban agglomeration in 2023 can be roughly divided into four categories. The first category includes Yibin and Luzhou, with a concentration range of 43.4–48.0 μg/m^3^. The second category includes Chengdu, Deyang, Leshan, Zigong, Neijiang, and Guang’an, with a concentration range of 38.80–43.40 μg/m^3^. The third category includes Chongqing, Nanchong, Mianyang, Meishan, and Ziyang, with a range of 34.20–38.80 μg/m^3^. The fourth category includes Dazhou, Suining, and Ya’an, with a range of 29.60–34.2 μg/m^3^. The heavily polluted areas are primarily located in the old industrial regions of southern Sichuan.
(4) The MSFRPM model assessed a total of 36 regional pollution events in the Chengdu-Chongqing Urban Agglomeration in 2023, with 14 instances of mild regional pollution events, 21 instances of moderate regional pollution events, and 1 instance of severe regional pollution event. Compared to the actual results, the assessment of regional PM_2.5_ pollution by the MSFRPM model closely aligns with the real outcomes, underestimating only one mild regional pollution event and one severe regional pollution event.

## Supporting information

S1 Data Data in the experiment. (ZIP)
